# Characterization of Extracellular Vesicles Isolated From Human Milk Using a Precipitation-Based Method

**DOI:** 10.3389/fnut.2020.00022

**Published:** 2020-03-13

**Authors:** Diana C. Bickmore, John J. Miklavcic

**Affiliations:** ^1^Food Science and Nutrition, Schmid College of Science and Technology, Chapman University, Orange, CA, United States; ^2^School of Pharmacy, Chapman University, Orange, CA, United States

**Keywords:** breastfeeding, dynamic light scattering, exosome isolation, exosome verification, fatty acids, nanovesicles, nanoparticle tracking analysis, scanning electron microscopy

## Abstract

Extracellular vesicles (EV) function in intercellular communication, and those in human milk may confer immunologic benefits to infants. Methods of EV isolation such as ultracentrifugation (UC) may not be feasible for the study of EVs in human milk due to the need for large sample volume. A technique to isolate EVs from a small volume of human milk using a precipitation reagent is described herein. Electron microscopy, nanoparticle tracking analysis, and semi-quantitative antibody array were conducted to confirm isolation of human milk EVs. Count, size, protein content, and fatty acid quantification of EVs were determined. This isolation technique yielded 8.9 x 10^9^ (± 1.1 × 10^9^) EV particles/mL of human milk. The present method meets the Minimal Information for Studies of Extracellular Vesicles (MISEV) guidelines. An established EV isolation method suitable for a low volume of human milk will facilitate further research in this growing area.

## Introduction

It is well-known that consumption of human milk is associated with enhanced infant health outcomes in comparison to consumption of infant formula. However, it is not fully known which components of human milk may be responsible for supporting optimal health and development of newborns. Increasing research suggests that EVs from human milk have physiologic function that may impact acute and chronic health outcomes. Human milk EVs promote epithelial cell growth in the intestine (1) and were found to protect intestinal epithelial cells from oxidative stress (2). Additionally, human milk EVs have been implicated in the immune modulating function of human milk, and may play a role in the development of the neonatal immune system (3). These effects may be attributed to the protein, lipid, or microRNA cargo of human milk EVs (3). A reliable method for consistent isolation of EVs from human milk is needed to determine the functional components of EVs to which enhanced infant health outcomes can be attributed.

Although UC is the most commonly used method to isolate EVs from biospecimens (4), the feasibility of this method for human milk research is limited. As a precious biofluid for feeding newborns, acquiring the large volume of human milk needed for EV isolation using UC is not always feasible. Unfortunately, no method of EV isolation has been authenticated for use with a low volume of human milk (≤2 ml); prior studies have isolated human milk EVs from a starting volume of 9 mL (5). As a result, the limitations in conducting research in this area have created a knowledge gap. Additionally, authentication of an EV isolation method from low volumes of human milk will facilitate research on EVs throughout milk production periods, the course of lactation, over time-of-day variation, and perhaps most importantly in low volume producers, which are not adequately studied.

This limitation has several potential negative consequences. First, analysis of only large volumes may limit research to use of pooled human milk. This measure would result in a greater understanding of average milk composition but not of interindividual variability. Second, analyses may be limited solely to time of lactation when higher volumes of milk are produced. This may then result in a disparate understanding of mature milk relative to early and transitional milks. Finally, research may be limited to studies of mothers with high volume of milk expression instead of low volume producers. Therefore, a strong need exists for a method of isolate EVs from a low volume of human milk.

A novel method for the isolation of EVs requires verification procedures. The International Society for Extracellular Vesicles (ISEV) released MISEV guidelines in 2018 (6) detailing the minimum criteria for confirming isolation of EVs. MISEV guidelines recommend that each EV preparation be (i) defined quantitatively by the source of EVs, (ii) characterized to determine the abundance of EVs by total particle number or protein/lipid content, (iii) tested for components associated with EV subtypes or EVs generically, and (iv) tested for the presence of non-vesicular co-isolated components. This paper describes a precipitation-based method for the isolation of EVs from human milk. The subsequent characterization of EVs suggest successful isolation in compliance with the MISEV guidelines. EVs isolated using the present method are therefore appropriate for downstream characterization and functional analyses to better understand the health and immune-modulating properties of human milk.

## Methods

**Figure 1:**
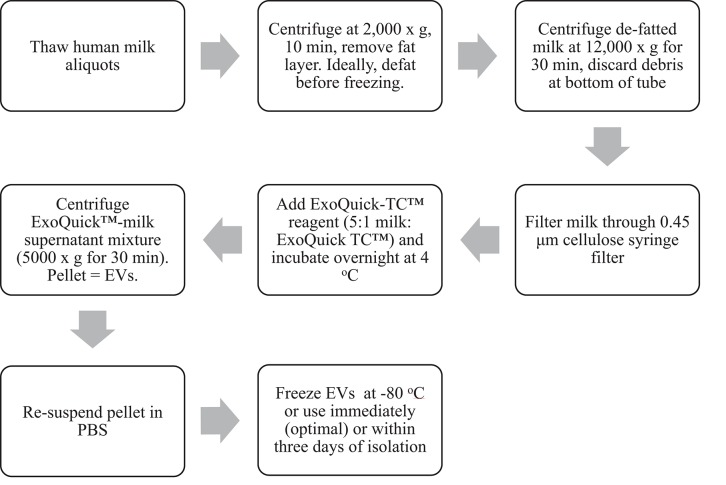
Workflow schematic of EV isolation from human milk using a precipitation-based isolation method.

Mature milk (>6 months after initiation of lactation) was pooled and pasteurized from donors to develop the EV isolation method (Prolacta Bioscience, City of Industry CA). Twelve volunteers also provided samples of expressed milk between 2 and 4 weeks postpartum which were immediately frozen at −80°C. Ethics approval was obtained from the Chapman University Institutional Review Board. The EV isolation method (Figure 1) outlined herein is adapted from the instructions for a commercial precipitation reagent (7) and a previously published protocol (8).

### EV Isolation Method

Thawing, Defatting, and Removal of Cell Debris

1. Thaw frozen human milk at 4°C. Once thawed, vortex milk for ~3 s.
2. If milk was not aliquoted into microcentrifuge tubes prior to freezing, aliquot 1.5–2 mL (or desired volume) human milk into microcentrifuge tubes.
3. Centrifuge at 2,000 × g for 10 min to separate and remove the fat layer with a metal spatula. Discard the fat layer and transfer milk to a new tube. Removing the fat layer also removes milk fat globules (9).
4. Centrifuge the defatted milk at 12,000 × g for 30 min to remove cell debris. Transfer milk supernatant and/or discard pellet.
5. Filter milk supernatant through a 0.45 μm cellulose syringe filter into a new microcentrifuge tube to further eliminate cells and cellular debris.

EV Isolation

6. Using a 5:1 ratio of milk supernatant: ExoQuick-TC™ reagent (System Biosciences, Palo Alto CA), add reagent to the filtered milk and gently invert until mixed.
7. Incubate at 4°C overnight or for at least 12 h.
8. After incubation, centrifuge at 5,000 × g for 30 min (beige pellet will appear at the bottom of the tube).
9. Discard supernatant, and resuspend EV pellet in 100–600 μL PBS (pH 7.4).
10. Depending on downstream application, use resuspended EVs stored at 4°C within 3 days or freeze immediately at −80°C.

### Scanning Electron Microscopy

Zeiss Gemini Sigma 300 scanning electron microscope (SEM) was used to visualize EVs isolated from milk expressed at 2 weeks postpartum (*n* = 1 volunteer). EVs were visualized 1 day after they were isolated, resuspended, and stored at 4°C. The original EV resuspension in PBS (500 μL) was further diluted in PBS (1:1,000). SEM slides were prepared with 2 μL of diluted EVs. Argon gas sputter coating of EVs with 3 nm gold-palladium alloy was performed to prevent sample destruction.

### Nanoparticle Tracking Analysis

Nanoparticle Tracking Analysis (NTA; Nanosight NS01) was used to determine the concentration and size of EVs isolated from the pooled milk sample. A sample of EVs originally resuspended in PBS (500 μL) and frozen at −80°C was thawed on ice and further diluted in PBS (1:75) prior to injection. Detection threshold was set to four, and three runs each of 30 s in duration were completed and analyzed using NTA 3.1 software. Total yield (EV particles/mL milk) was calculated based on dilution factors and a starting volume of 1.5 mL milk.

### Dynamic Light Scattering

The diameter of EVs isolated from the pooled milk sample was measured with a Mobius Dynamic Light Scattering (DLS) instrument (Wyatt Technology) using DLS Firmware Version 1.2.0.0. Laser wavelength was set to 532 nm, and a detector angle of 163.5° was used. DLS acquisition time was set to 5 s and a number acquisition of three was used to perform three technical replications on EVs stored at 4°C over the course of 10 days.

### Exocheck Antibody Array

The Exocheck™ Antibody Array (System Biosciences, Palo Alto CA) was used according to the manufacturer's instructions (10) to determine the presence or absence of common EV proteins (CD63, EpCAM, Annexin5, TSG101, Flotilin1, ICAM, ALIX, CD81) in EVs isolated from milk expressed at 4 weeks postpartum (*n* = 1 volunteer). Resuspended EVs were thawed on ice prior to antibody array analysis.

### Determination of Total Fatty Acid Concentration

The EVs from which fatty acids were analyzed were isolated using 2 mL aliquots of pooled milk, and with variations in EV isolation steps. A 5:1 and 10:1 ratio of milk supernatant: ExoQuick-TC™ reagent was used with or without (0.45 μm cellulose) filtration or purification using ExoQuick-TC™ ULTRA purification columns according to the manufacturer's instructions (System Biosciences, Palo Alto, CA). Prior to fatty acid analysis, EVs were isolated from the pooled milk sample, resuspended in PBS (500 μL), frozen at −80°C, and thawed on ice. Fatty acid analysis was performed by Creative Biostructure (Shirley, NY USA).

The total fatty acid concentration of EVs was determined by colorimetric analysis in triplicate (*n* = 1 per isolation variation). Standards were prepared with palmitic acid (1 nmol/μL). Samples were diluted and homogenized. Standard dilution (50 μL) or sample (0.5–25 μL) were added to each sample well. The final volume was adjusted to 50 μL with assay buffer. An acyl-coenzyme A synthetase reagent (2 μL) was added to each reaction well, mixed, and incubated (20 min, 37°C). Samples were then incubated (30 min, 37°C) in the dark with reaction mix (2 μL) containing assay buffer (44 μL), fatty acid probe (2 μL), enzyme mix (2 μL), and enhancer (2 μL). Finally, optical density was measured on a microplate reader at 562 nm.

### Protein Quantification

A Qubit™ 4 Fluorometer was used to measure the protein concentration in human milk EVs isolated from milk expressed at 2 weeks postpartum (*n* = 10 volunteers). Resuspended EVs were thawed on ice prior to protein quantification. The instrument was calibrated with protein standards according to the manufacturer's instructions (11). EV samples originally resuspended in 600 μL PBS were thawed on ice and diluted in PBS (1:20). Lysis buffer (10 μL) was added and samples were vortexed (Protease Inhibitor Cocktail, RIPA buffer, Thermo Fisher Scientific, Waltham MA). Protein concentration was measured in duplicate after incubating (15 min, room temperature) the lysate (1 μL) with working reagent (199 μL). Protein quantification of EVs was calculated based on dilution factors and a starting volume of 1.5 mL milk.

## Results

**Figure 2:**
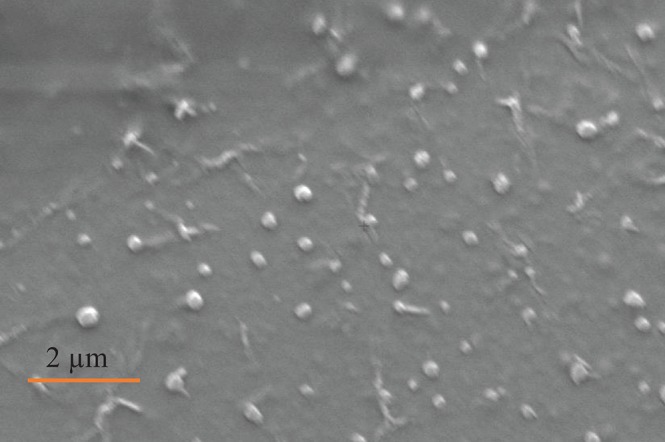
Image of EVs obtained by SEM from participant (*n* = 1), Electron high tension = 5.00 kV, working distance = 20.9 mm, detector = secondary electron, magnification = 8.70 K X, vacuum mode = high vacuum, height = 9.851 μm.

**Figure 3:**
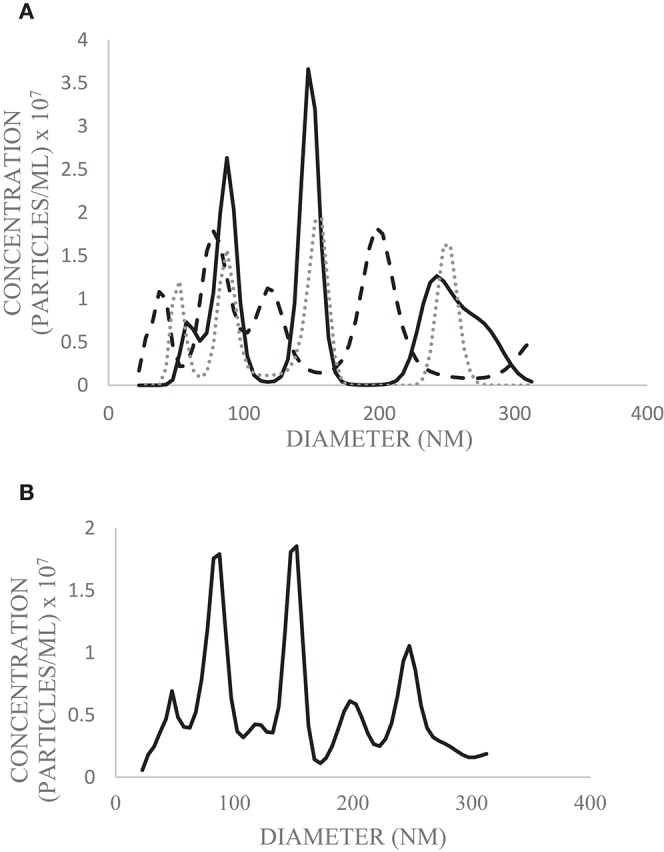
Diameter and concentration of human milk EVs from pooled human donor milk measured by NTA. Technical replicates were performed in triplicate (solid line = trial 1; dashed line = trial 2; dotted line = trial 3) **(A)** and the average of the three runs was calculated **(B)**. The above graphs are plotted from the 10th−90th percentile of EV sizes (22.5–312.5 nm) to exclude particles which do not meet the size criterion for likely EVs.

**Figure 4:**
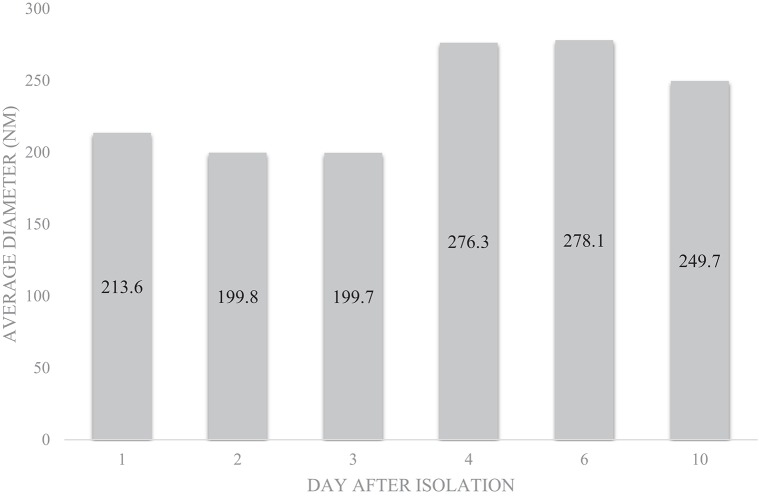
Average diameter of EVs from pooled human donor milk measured for 10 consecutive days after isolation and storage at 4°C. Error bars were excluded because individual standard deviations were <5% of the mean. Pooled SD = 33.45.

**Figure 5:**
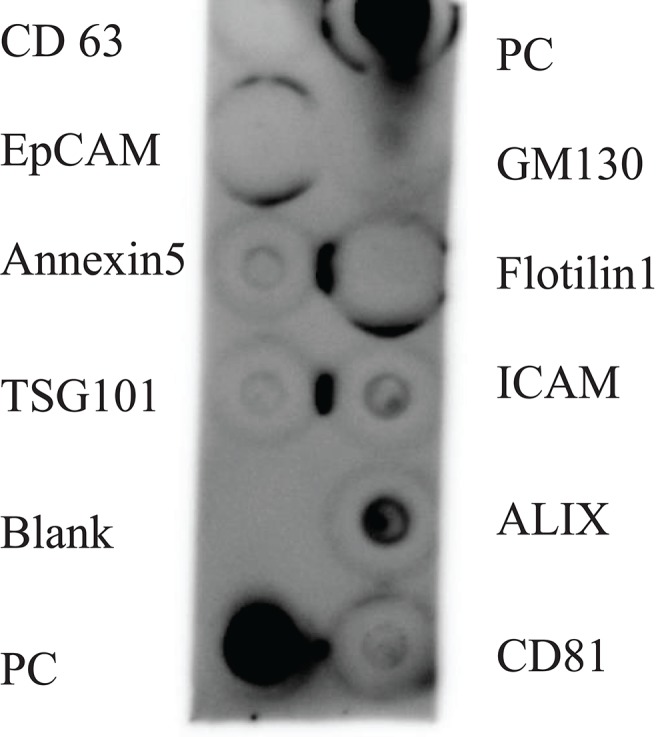
Antibody array of human milk EVs from participant (*n* = 1). PC represents the positive control, and GM130 is a cellular contamination marker. CD 63 = cluster of differentiation 63, EpCAM = epithelial cell adhesion molecule, TSG101 = tumor susceptibility gene 101, ICAM = intracellular adhesion molecule, ALIX = ALG-2-interacting Protein X, CD81 = cluster of differentiation 81.

SEM (Figure 2), NTA (Figure 3), DLS (Figure 4), and an antibody array (Figure 5) were used to image, quantify, measure the average diameter, and identify protein markers characteristic of EVs. The image obtained by SEM (Figure 2) revealed the size of nanovesicles in the expected range for EVs, approximately 50–350 nm. Results from analysis by NTA (Figure 3, Supplementary Figure 1, Video 1) revealed that the isolation method yielded 8.9 × 10^9^ (± 1.1 × 10^9^) particles/mL of human milk. The mean and mode diameter of EVs were 179.3 and 150.3 nm, respectively (Figure 3). No standard deviation is reported for the mean since a trimodal distribution of EV populations was observed. Results from DLS (Figure 4) showed that the average diameter of EVs 1 day after isolation was 213.6, and 249.7 nm 10 days after isolation. Error bars for individual days were excluded because individual standard deviations for technical replicates were <5% of the mean. Antibody array (Figure 5) indicated that the sample was positive for the following known EV markers: cluster of differentiation 81 (CD81), ALG-2-interacting Protein X (ALIX), intracellular adhesion molecule (ICAM), tumor susceptibility gene 101 (TSG101), and Annexin5, and negative for cluster of differentiation 63 (CD63), epithelial cell adhesion molecule (EpCAM), and flotilin1.

**Figure 6:**
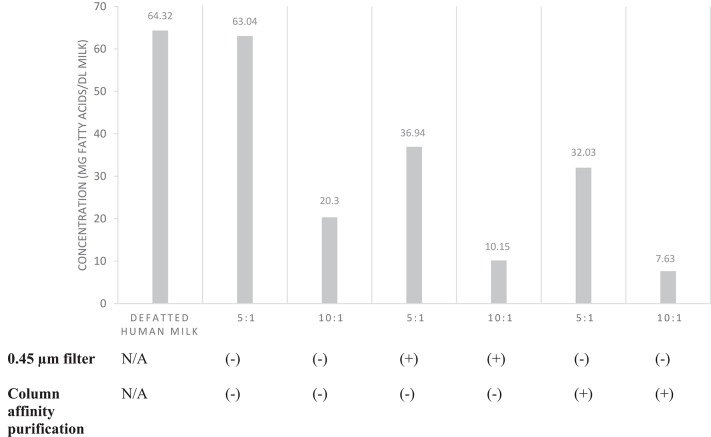
Total fatty acid concentration of EVs isolated from pooled human donor milk. The ratios represent the proportion of milk supernatant: ExoQuick-TC™ reagent used during isolation. Milk was either filtered (0.45 μm) prior to EV isolation or EVs isolated were purified by column affinity after isolation. Error bars were excluded because individual standard deviations for two technical replicates were <5% of the mean. Pooled SD = 18.78.

After verification of isolation, human milk EVs were characterized by quantifying total fatty acids (Figure 6) and protein concentration (Table 1). The average total fatty acid concentration of EVs isolated with the recommended method (5:1, filter, no column purification) was 36.94 mg/dL. The mean protein concentration of human milk EVs was 5.08 (±0.15) mg/dL.

**Table 1:** Protein concentration of EVs isolated from human milk (*n* = 10).

**Sample**	**Protein content (mg/dL milk)**
1	5.20
2	5.00
3	5.11
4	5.02
5	5.15
6	5.18
7	5.24
8	4.98
9	5.20
10	4.75
Mean	5.08
SD	0.15

*For each sample, technical replicates were performed in duplicate*.

## Discussion

The method of EV isolation from human milk described herein meets the MISEV criteria (6) for verifying the presence of EVs. EVs isolated with the proposed method were (i) quantified in relation to the source of human milk, (ii) characterized to determine the abundance of EVs by total particle number and lipid & protein content, (iii) tested for the presence of markers associated with EVs, and (iv) tested for the presence of non-vesicular co-isolated components. The method adapted from manufacturer instructions for a precipitation reagent (7) and previous literature (8) was shown to be suitable to adequately characterize EVs isolated from human milk and for downstream applications.

There is consistency between the average EV diameter measured by SEM, NTA, and DLS (Figures 2–4). Unlike NTA which generates size distribution data, DLS measures the average particle diameter. Measurement by DLS then may be skewed by low concentrations of outliers or clustering of particles (12). Therefore, the ~15% difference in diameter between SEM, NTA, and DLS measurements could be due to overestimation of diameter by DLS. The recommended method presented herein yielded 8.9 × 10^9^ (± 1.1 × 10^9^) EV particles/mL of human milk. Another group isolated human milk EVs and reported a yield of 8.0 × 10^10^ particles/ml of milk using a UC based method (5). The difference in yield could be attributed to the fact that banked, pasteurized milk was used in the present method. Additionally, EVs were frozen and thawed prior to quantification without defatting before initial freezing, which has shown the decreased recovery of EVs (3).

In the MISEV guidelines, it is recommended that operational terminology for extracellular vesicles based on factors such as size be used. EVs <200 nm in diameter would be considered “small,” and EVs >200 nm considered medium or large (6). Results from NTA indicated that the greatest concentration of particles is around 153 nm (Figure 3), meaning the EV population in highest abundance would be classified as small. The 10th−90th percentile of particle size were graphed (Figure 3), as particles outside this range were likely aggregates or fragments.

Because storage conditions may affect EV characterization, MISEV guidelines indicate the importance of describing storage conditions such as storage container, temperature, buffer, freeze-thaw cycles of biofluid and EVs, etc. (6). It was previously found even that storage of EVs for 2 h at 4°C decreased the viability of the exosome population, but the change in size was not measured (3). The timecourse experiment (Figure 4) represents storage-induced changes in diameter starting from freshly isolated EVs measured over the course of 10 days. The average diameter of EVs measured by DLS increased over time after isolation and storage. This may indicate swelling and enlargement of EVs, or aggregation of particles. Therefore, when performing studies to determine the relation between structure and function, it may be advantageous to use EVs immediately after isolation.

For protein-based verification of EV isolation, MISEV guidelines stipulate that at least one type of protein in two broad categories should be positively identified and the absence of one negative marker indicated. These categories include transmembrane or GPI-anchored proteins, such as the tetraspanins CD63 and CD81, and EV-recovered cytosolic proteins such as ALIX and flotillins-1 and 2. To verify the absence of non-EV isolated co-structures, markers such as albumin can be used (6). The antibody array (Figure 5) verified that human milk EVs isolated were positive for proteins in the tetraspanin and EV-recovered cytosolic proteins category, and also negative for cellular contamination marker.

The amount of exosomal protein has been used as a means of EV quantification (13). Considering that the average protein concentration measured in EVs was 5.08 mg/dL, EV protein comprises ~0.42% of total protein from mature human milk, assuming the protein concentration of mature human milk is ~1,200 mg/dL (14). However, it should be noted that protein quantification with biofluids such as human milk may not be a consistent and reliable method of quantification due to the presence of co-isolated molecules. Therefore, we reported the total fatty acid concentration of human milk EVs (Figure 6). Based on the assumption that fat content of human milk is primarily in the form of triglyceride, we estimated that EV fatty acids are ~0.8% of total fatty acids in mature human milk (14).

We compared fatty acid quantification among EVs isolated from human milk with different volumes of reagent, use of size exclusion filter, and with or without column affinity purification. We suggest a supernatant-to-precipitation reagent ratio of 5:1 for optimal yield of EVs to quantify fatty acids. We also suggest filtration of milk by size exclusion after defatting to remove non-EV artifacts such as casein and cellular debris. However, it is unclear whether column affinity purification after EV isolation performs similarly to size exclusion filtration of milk supernatant prior to EV isolation. Although fatty acid quantification was similar after each method, it is unknown if filtration and purification result in differences in the EV populations isolated.

The present method of isolating EVs from human milk fulfills the MISEV criteria by characterizing the EVs with quantitative and qualitative methods, confirming the presence of characteristic EV markers, and confirming (Figure 5) the absence of non-EV components. The application of this isolation method extends beyond the applications detailed in our manuscript. The ability to successfully isolate EVs from small volumes of human milk can be applied to miRNA isolation, proteomics, lipidomics, and functional *in vitro* assays.

## Conclusions

EVs were successfully isolated from human milk using a precipitation reagent. The method yielded 8.9 × 10^9^ ± 1.1 × 10^9^ EV particles/mL of human milk. Protein and fatty acid concentration of EVs in human milk were determined and the percentage of fatty acids and protein in EVs relative to the whole milk were ~0.8% and ~0.42%, respectively. The method presented is consistent and reliable for isolating, quantifying, and characterizing human milk EVs for research and clinical purposes and in continuing to understand the human milk food matrix. As a dynamic food and biofluid, future study may elucidate how EVs vary over i) early, transitional and mature milk production periods, ii) course of lactation (fore vs. hind milk), and iii) time-of-day variation. This method can be used to elucidate the role of human milk EVs in neonatal health and immune system development, and for applications of formula and human milk fortifier production.

## Data Availability Statement

The datasets generated for this study are available on request to the corresponding author.

## Ethics Statement

The studies involving human participants were reviewed and approved by Chapman University IRB. The patients/participants provided their written informed consent to participate in this study.

## Author Contributions

DB isolated the EVs from human milk, performed all experiments with the exception of NTA and total fatty acid analysis, and drafted the manuscript. JM oversaw the writing of the manuscript, obtained funding for the study, and collected NTA data.

### Conflict of Interest

The authors declare that the research was conducted in the absence of any commercial or financial relationships that could be construed as a potential conflict of interest.

## Supplementary Material

Video 1 Human milk EVs from NTA light scatter using Nanosight NS01. Video is a representative segment captured from a 3 × 30 s flux at infusion of 40 μL/min into microfluidics chamber.
