# Supplementary material for: Characterization of Extracellular Vesicles Isolated From Human Milk Using a Precipitation-Based Method
Source: Front Nutr. 2020 Mar 13;7:22. doi: 10.3389/fnut.2020.00022 (PMC7082312; doi:10.3389/fnut.2020.00022)
Supplement: Supplementary file 1 [file Image_1.pdf]

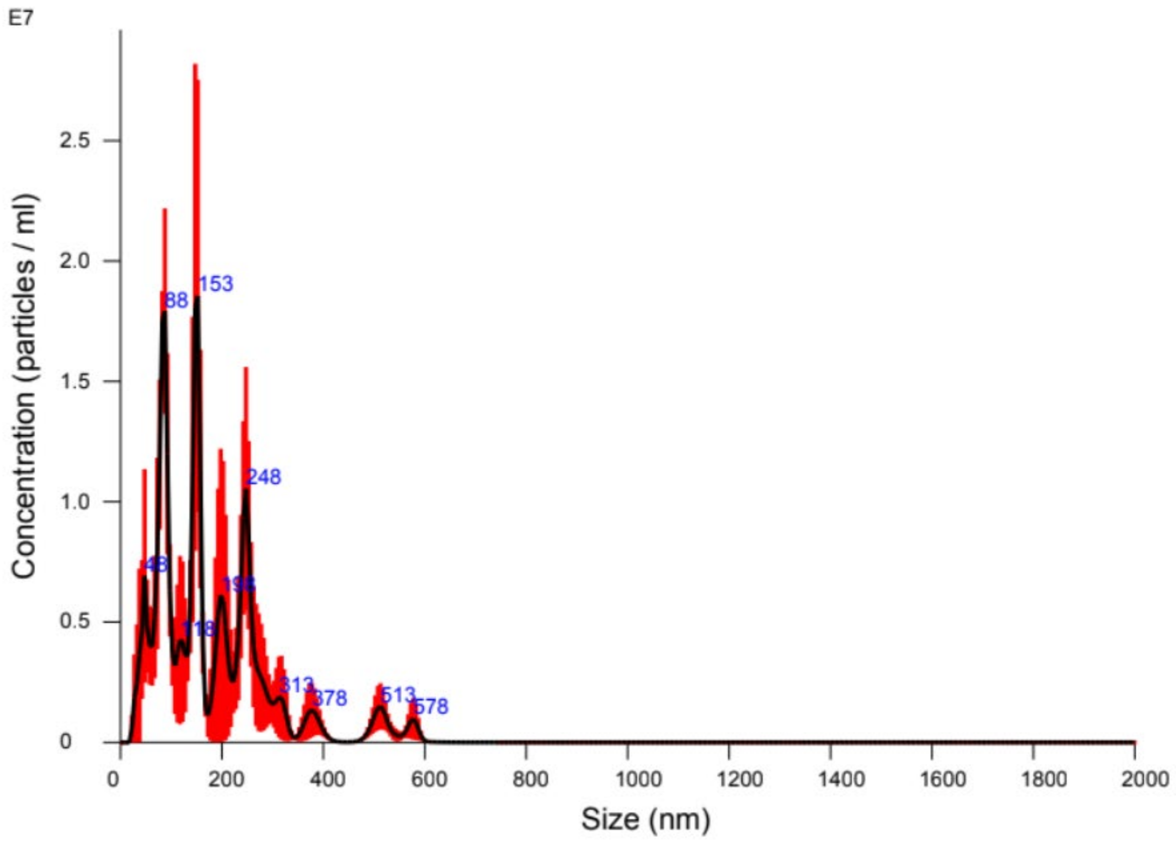

Supplementary Figure 1. Size distribution graph from nanoparticle tracking analysis (Nanosight NS01). Graph is the average of 3 x 30 sec flux at infusion of 40 μL/min into microfluidics chamber. Error bars indicate + / - 1 standard error of the mean.
